# Left-Sided Inferior Vena Cava Encountered During Organ Retrieval Surgery: Report of Two Cases

**Published:** 2016-11-01

**Authors:** Y. Rajabnejad, M. Aliakbarian, A. Rajabnejad, M. R. Motie

**Affiliations:** Surgical Oncology Research Center, Mashhad University of Medical Sciences, Mashhad, Iran

**Keywords:** Vena cava, inferior, Anatomic variation, Tissue and organ harvesting, Transplantation, Liver

## Abstract

Left-sided inferior vena cava (IVC) is the second most common anatomical anomaly of the IVC after duplication. Herein, we present two cases of left-sided IVC, diagnosed during organ retrieval procedure. In a young brain-dead man, a single left-sided IVC was observed; it originated from iliac confluence in the left side of the aorta and extended throughout the abdomen. There was no retrohepatic IVC in the patient; hepatic veins drained directly into the right atrium. The second case was a brain-dead young woman with a left-sided IVC originated from iliac confluence to the kidney level; then, the IVC crossed anterior to the abdominal aorta to join a normally positioned retrohepatic IVC. In cases of retroperitoneal surgeries, IVC anomalies should be considered during preoperative imaging studies, because they may be misdiagnosed as para-aortic lymphadenopathy, tumor or dilated gonadal vein that may result in iatrogenic damage during surgery.

## INTRODUCTION

Inferior vena cava (IVC) anomalies are infrequent, but of high importance in some urological, vascular, and transplant surgeries. Left-sided IVC, the second most common anomaly after duplication, with an incidence rate of up to 3% is observed in about 0.04%–0.5% of population [1-4]. This anomaly is usually found incidentally on imaging studies including computed tomography (CT) or magnetic resonance imaging (MRI) performed for other reasons [3]. A careful assessment of these anomalies may prevent iatrogenic injuries. In addition, keeping it in mind allows the transplant surgeon to be prepared for such a situation. Herein, we present on two cases of left-sided IVC, diagnosed during organ retrieval procedure.

## CASE REPORT

Case 1

**Figure 1:**
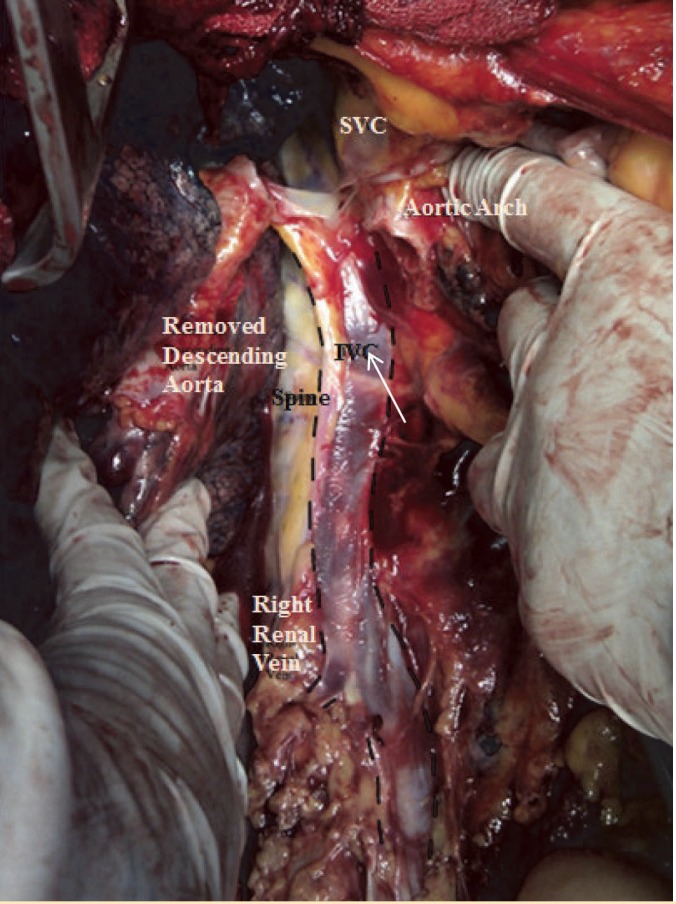
Anterior view of the abdominal and thoracic inferior vena cava. Note malposition of the inferior vena cava in the left side of the spine and aorta

**Figure 2:**
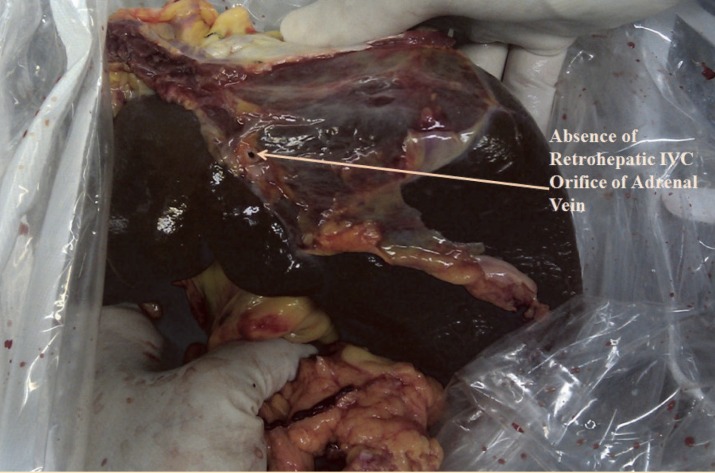
Inferior view of the liver. Note the absence of retrohepatic vena cava

A 25-year-old man became brain-dead following a severe blunt head trauma was admitted to our hospital for organ donation. Surgery was performed with a median sternotomy and a midline laparotomy. During surgery a single left-sided IVC was observed; it originated from the confluence of the left and right common iliac veins and ascended vertically to the left side of the abdominal aorta and anterior to the ureter, receiving the right renal vein passing anterior to the aorta (Fig 1). Expectedly, the left renal vein was shorter than normal. The IVC was in the left side of the aorta throughout the abdomen. After entering the thorax, the passage of IVC was posterior to the aorta toward the right atrium. There was no retrohepatic IVC in the case and drainage of hepatic veins were directly into the right atrium (Fig 2). No associated anomaly was noted in this case. 

Case 2

During laparotomy for organ retrieval surgery on a 36-year-old woman with brain death secondary to head trauma, we encountered to a left-sided IVC. It originated from the confluence of the right and left iliac veins at the left side of the aorta. After ascending to the kidney level, the left renal vein joined the left-sided IVC, which crossed anterior to the abdominal aorta to join a normally positioned retrohepatic IVC. The anatomy was normal along the path of the IVC. 

## DISCUSSION

IVC anomalies occur in the complex process of embryogenesis, which takes place in between the sixth to tenth weeks of gestation [5]. These anomalies include: left-sided IVC, double inferior vena cava, retroaortic left renal vein, circumaortic renal collar, and periureteral venous ring. In the embryogenesis of the IVC, three pairs of venous systems are involved: posterior, subcardinal, and supracardinal veins. Naturally, IVC is formed by the right supracardinal vein. In the case of left-sided IVC, the left supracardinal vein persists and the right vein regresses [6]. However, different regression degrees of right supracardinal vein happen among cases of left-sided IVC [7]. Khamanarong dissected and examined 939 cadavers and found only one case of this anomaly [8]. Left-sided IVC or transposition of the IVC is a very unusual event, but is not incompatible with a normal life [8]. Most of the anomalies of the IVC are asymptomatic. However, cases of venous thrombosis and pulmonary embolism due to IVC anomaly, have been reported in the literature [3, 9, 10]. Caval anomalies are often discovered at venographic examinations, during operation or postmortem. 

Caval anomalies are important in retroperitoneal surgeries because they may be misdiagnosed as para-aortic lymphadenopathy, tumor, or dilated gonadal vein. So it could cause iatrogenic damage to the vein and result in hemorrhage [3, 8, 11]. Also, it is likely to complicate the left retroperitoneal approaches to the abdominal aortic aneurisms. Therefore, a transperitoneal approach would be preferable when the IVC is left-sided. This anomaly may complicate organ retrieval surgery as well. As there is no time to fully investigate the probable anomalies in a deceased donor, it is usually encountered during operation and may complicate the organ retrieval procedure or even damage the organs in unexperienced hands.

The typical endpoint of a left-sided IVC is at the level of left renal vein, where it crosses anterior to the aorta to form a normal right-sided IVC [12]. However, in one of our cases, the IVC did not cross the aorta and continued its passage upward. 

Although there was no retrohepatic IVC, this issue was not a contraindication for liver transplantation and the liver of the patient was successfully transplanted. However, the recipient should be selected carefully because when the surgeons use a graft without retrohepatic IVC, they must keep the recipient retrohepatic IVC. In other words, the piggy-back technique of hepatectomy is the only choice and using the standard technique is impossible.

Contrast-enhanced CT can be very useful for accurate pre-operative evaluation of retroperitoneal venous system anatomies and probable variations. MRI is also helpful, but it is expensive and time-consuming [1, 13]. The accurate pre-operative evaluation of these anomalies allows surgeons in selecting the most appropriate operation strategy to avoid probable iatrogenic damages.

In conclusion, left-sided IVC is a rare anatomical variation that has significant surgical implications. Preoperative identification with imaging modalities is critical in preventing unexpected surgical complications.
